# Supplementary material for: Influence of tectonics on global scale distribution of geological methane emissions
Source: Nat Commun. 2020 May 8;11:2305. doi: 10.1038/s41467-020-16229-1 (PMC7210894; doi:10.1038/s41467-020-16229-1)
Supplement: Supplementary file 1 — Supplementary Information [file 41467_2020_16229_MOESM1_ESM.pdf]

Supplementary Information

Influence of tectonics on global scale distribution of geological methane emissions

G. Ciotoli et al.

Supplementary Note 1 : Geo-CH_4 seepage definitions

Natural gas seepage consists of surface fluid manifestations (macro-seeps) and diffuse exhalation (microseepage) of hydrocarbons (mainly methane, subordinately ethane, propane, butane) in petroleum sedimentary basins[1,2] generally directly connected to reservoirs and source rocks within a petroleum system[3] (Supplementary Figure 1). Macro-seeps, defined and described in detail, by [2,4,5] include:

- gas seeps: surface manifestations that release only a gaseous phase. Gas may vent from outcropping rocks, through the soil horizon, or through river/lake beds.
- oil seeps: surface manifestations that release mainly crude oil, often associated with variable amounts of a gaseous phase. The amount of gas in oil seeps decreases during oil exposure to the atmosphere, with subsequent oxidation, biodegradation, and solidification. Asphalts and tars (solid seeps) do not generally contain significant quantities of gas.
- mud volcanoes: cone shaped structures produced over faults by the upwelling of sediments (mud) fluidised by gas and water (sedimentary volcanism); they may develop as single isolated cones and craters or, more frequently, as groups of cones and crater systems. The diameter of single craters may range from a few cm to several tens of meters and conical structures can be several hundreds of meters high.

Water springs with high concentrations of methane and other hydrocarbons in solution can also be considered a form of seeps[2]. However, we did not include them in the present study since the spatial distribution of a spring can be strongly controlled by non-tectonic hydrogeological factors (hydrologic circuit, outcrops of conductive, permeable strata).

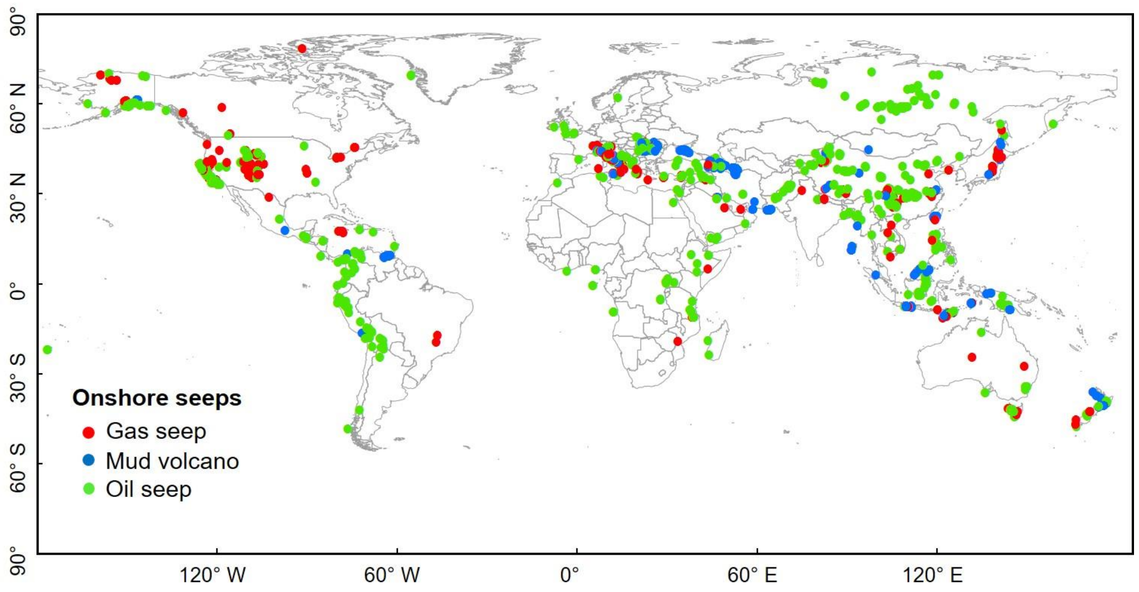

Supplementary Figure 1: Global distribution of onshore hydrocarbon seeps. Seeps distribution is based on the inventory of Etiope et al. (2019)[6].

Supplementary Note 2 : Conceptual workflow

We used geospatial analysis to explore statistical and spatial distribution (i.e., proximity analysis) of global data, to transform vector data in grid data (i.e., fault density maps) and to reclassify the maps of the considered geological factors (such as global sedimentary basins, petroleum fields, faults database and heat flow data) in the form of Boolean maps 0/1. We then jointly analysed these factors using the weighted overlay technique of Spatial (GIS-based) Multi-Criteria Decision Analysis (SMCDA) in order to create a model of favourability. The flowchart summarizes the relationships among the datasets, the applied techniques of geospatial analysis and the obtained results (Supplementary Figure 2).

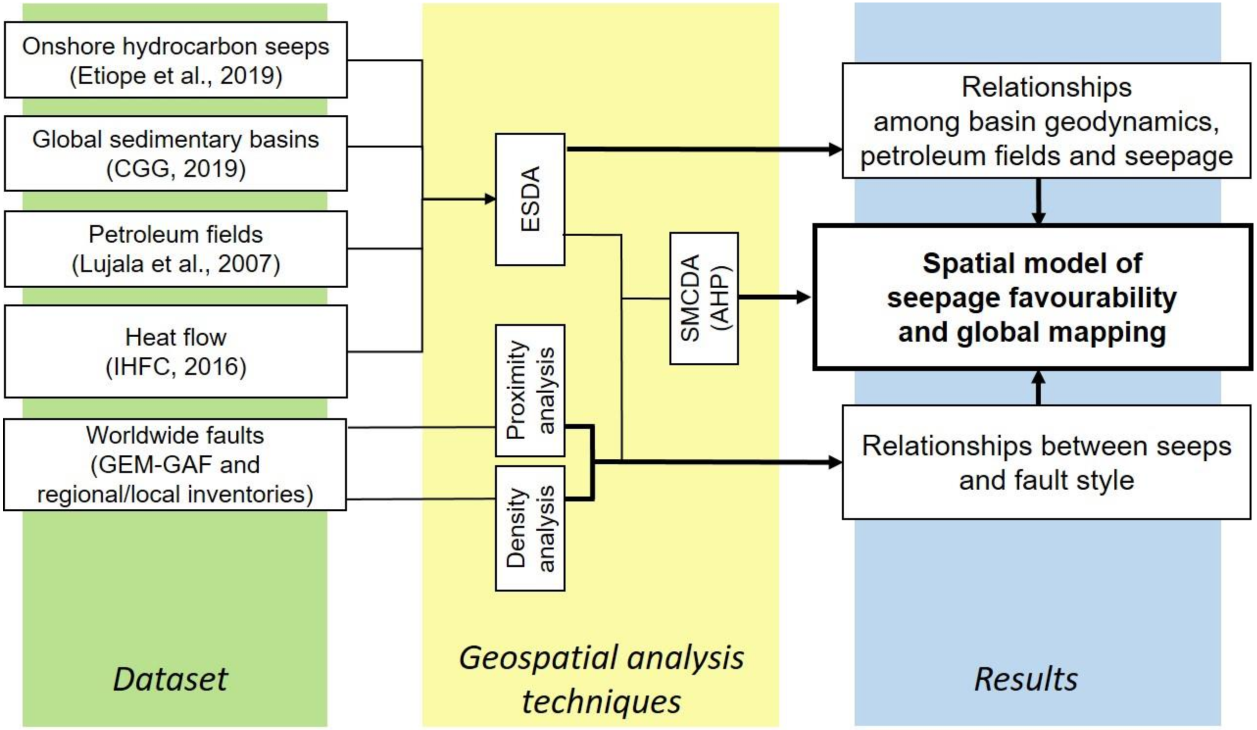

Supplementary Figure 2: Logical flow scheme of the geospatial analysis. ESDA, Exploratory Spatial Data Analysis; SMCDA, Spatial Multi-Criteria Decision Analysis; AHP, Analytic Hierarchical Process.

Supplementary Note 3 : Further relationships among seeps, sedimentary basins, petroleum fields and heat flow

We used the classification of sedimentary basins (convergent and divergent, including wrench basins) according to the Sedimentary Basins World Map from http://www.datapages.com/associatedwebsites/gisopenfiles/robertstellussedimentarybasinsoftheworldmap.aspx. Convergent basins and orogenic deformations are related to the development of subduction systems along active continental margins or island arcs, including:

-Peripheral Foreland, Intramontane Wrench, Late to Post Orogenic Extension and Trapped Oceanic Crustal Sag (subduction by continent-continent collision);

- Fore-arc, Retroarc Foreland, Retroarc Extensional and Arc-related Wrench (subduction ocean-continent and ocean-ocean suture).

Divergent basins are dominantly extensional in character and commonly form in either mantle-generated or lithosphere-generated rift zones. Individual basins frequently undergo an evolution from syn-rift phase, with active block rotation, to a post-rift phase where more broadly based subsidence is controlled by thermal cooling. Divergent basins include: Wrench, Passive Margin, Intracratonic Wrench, Post-rift sag, Rift and Intracratonic Sag. Supplementary Figure 3a shows the total percentage of seep types occurring within the sub-groups of convergent and divergent basins.

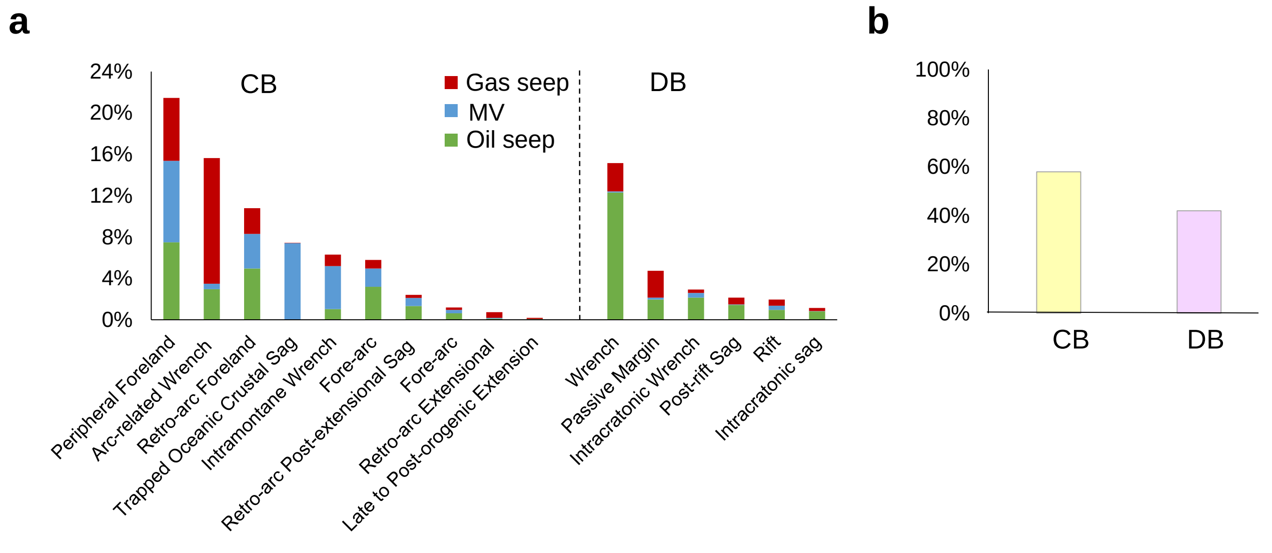

Supplementary Figure 3: Distribution of seeps within sedimentary basins. (a) Bar chart of the percentage distribution of seep types within the sub-regime types of sedimentary basins; CB= convergent basin, DB= divergent basin; (b) bar chart of the percentage of petroleum fields in convergent and divergent basins.

Sedimentary basins are intersected with petroleum field dataset by using the Intersect tool of ArcGIS Pro. The results show that 98% of the petroleum fields occur in areas covered by sedimentary basins: 58% (517) in convergent basins and 42% (374) in divergent basins, respectively (Supplementary Figure 3b).

Supplementary Figures 4a and 4b show two examples, from California (USA) and China respectively, of seep distribution at the margins of petroleum fields, which are typically more faulted and fractured[1,5].

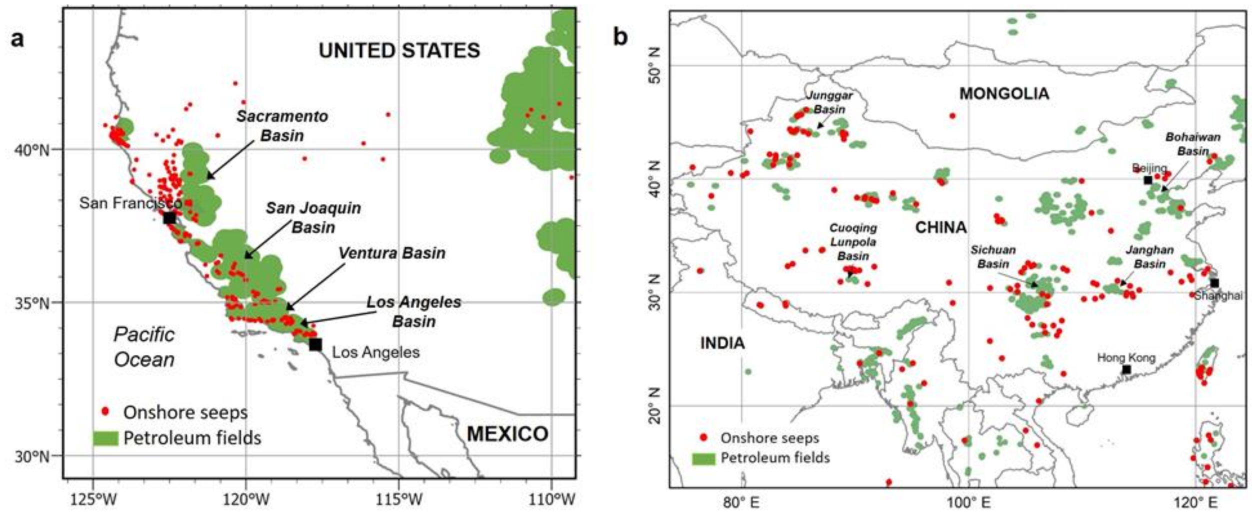

Supplementary Figure 4: Examples of seep distribution at the margin of petroleum fields. (a) Sacramento, San Joaquin, Ventura and Los Angeles basins (USA), (b) Sichuan, Janghan, Junggar, Cuoqing Lunpola and Bohaiwan basins (China).

The relationship between seep occurrence and heat flow was analysed by extracting heat flow values (mW m^{-2}) at seep locations. The map of the global heat flow was built from the Global Heat Flow Database of the International Heat Flow Commission (2016)[7] by using kriging interpolation (Supplementary Figure 5).

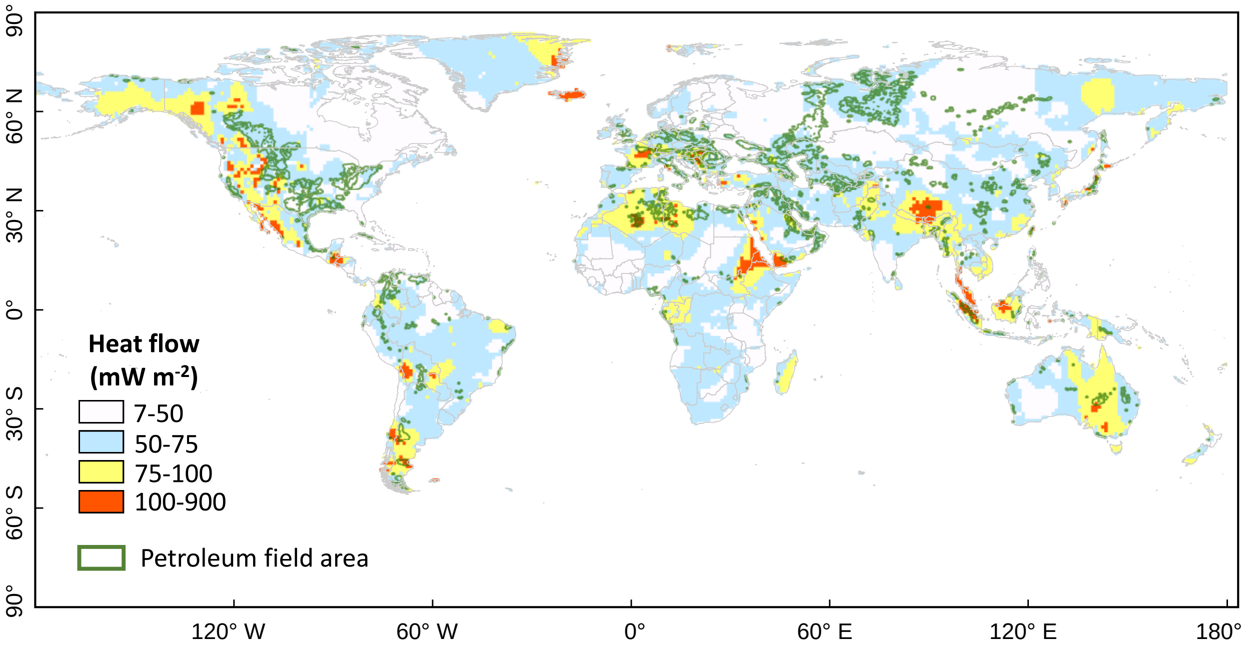

Supplementary Figure 5: Onshore heat flow map. Input data are from the Global Heat Flow Database[7].

The statistical analysis of the heat flow data extracted at each seep location indicates that seeps do not occur in areas with heat flux > 145.5 mW m^{-2}. Most of seeps (95%) occurs in areas with heat flow < 98.2 mW m^{-2}. The mean heat flux value at seep locations is 62.1 mW m^{-2}. In particular, gas seeps show the highest mean value (72.3 mW m^{-2}) (Supplementary Table 1).

Supplementary Table 1. Main statistics of heat flow values (mW m^{-2}) extracted at seep locations. Min: minimum value; Max: maximum value; LQ: lower quartile; UQ: upper quartile; 5%: 5th percentile; 95%: 95th percentile; Std. Dev.: standard deviation.

Type	N	Mean	Min	Max	LQ	UQ	5%	95%	Std. Dev.
Total seeps	2699	62.1	11.1	146.5	48.9	71.6	34.4	98.2	19.9
Oil	1119	60.9	11.1	146.5	51.9	71.6	40.2	82.0	17.0
Gas	839	72.3	24.4	145.8	55.2	96.7	44.0	100.4	23.0
MV	741	52.5	29.1	128.3	43.1	58.8	37.5	68.3	14.0

Supplementary Note 4 : Seeps and faults

Fault density map. Supplementary Figure 6 shows the global distribution of faults included in the new database assembled and edited for this study (see Data Availability).

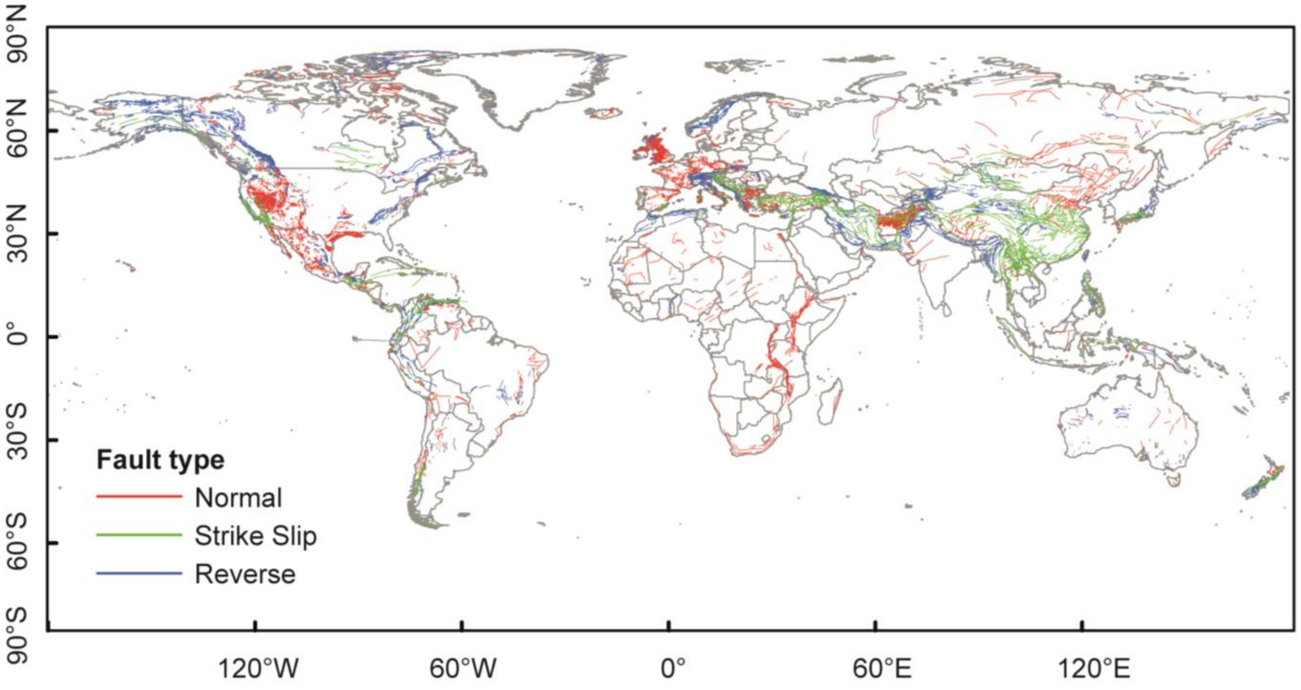

Supplementary Figure 6: Map of the global fault dataset.

Examples of regional maps extracted from the global dataset are reported below (Supplementary Figures 7a-e)

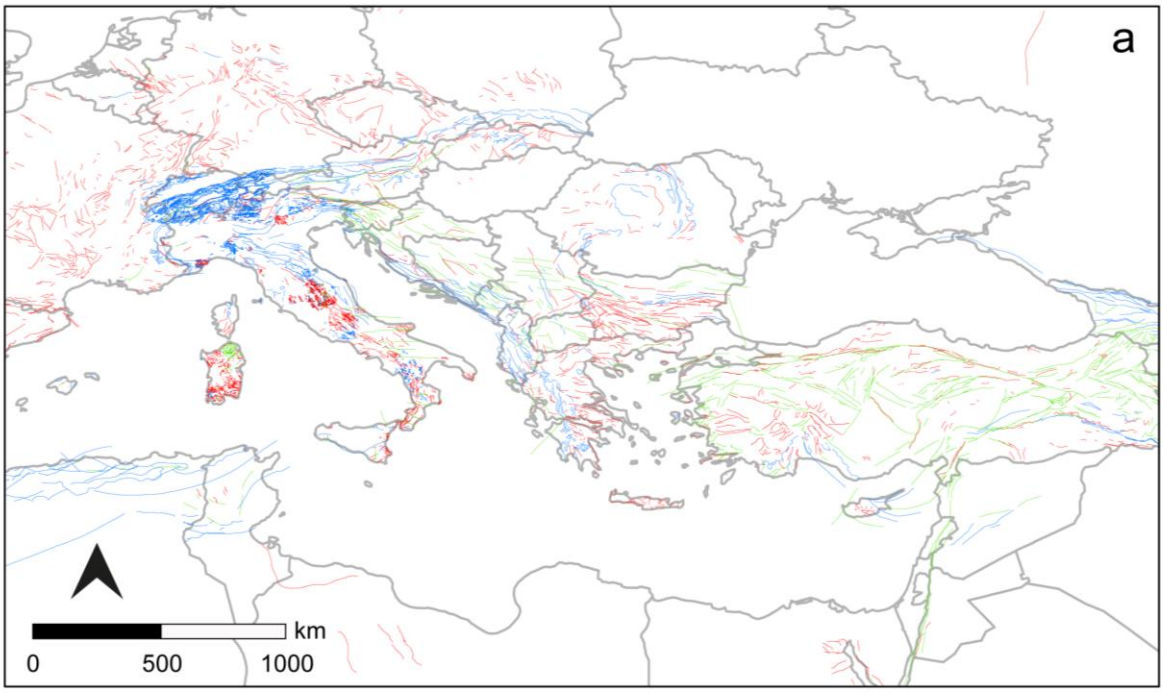

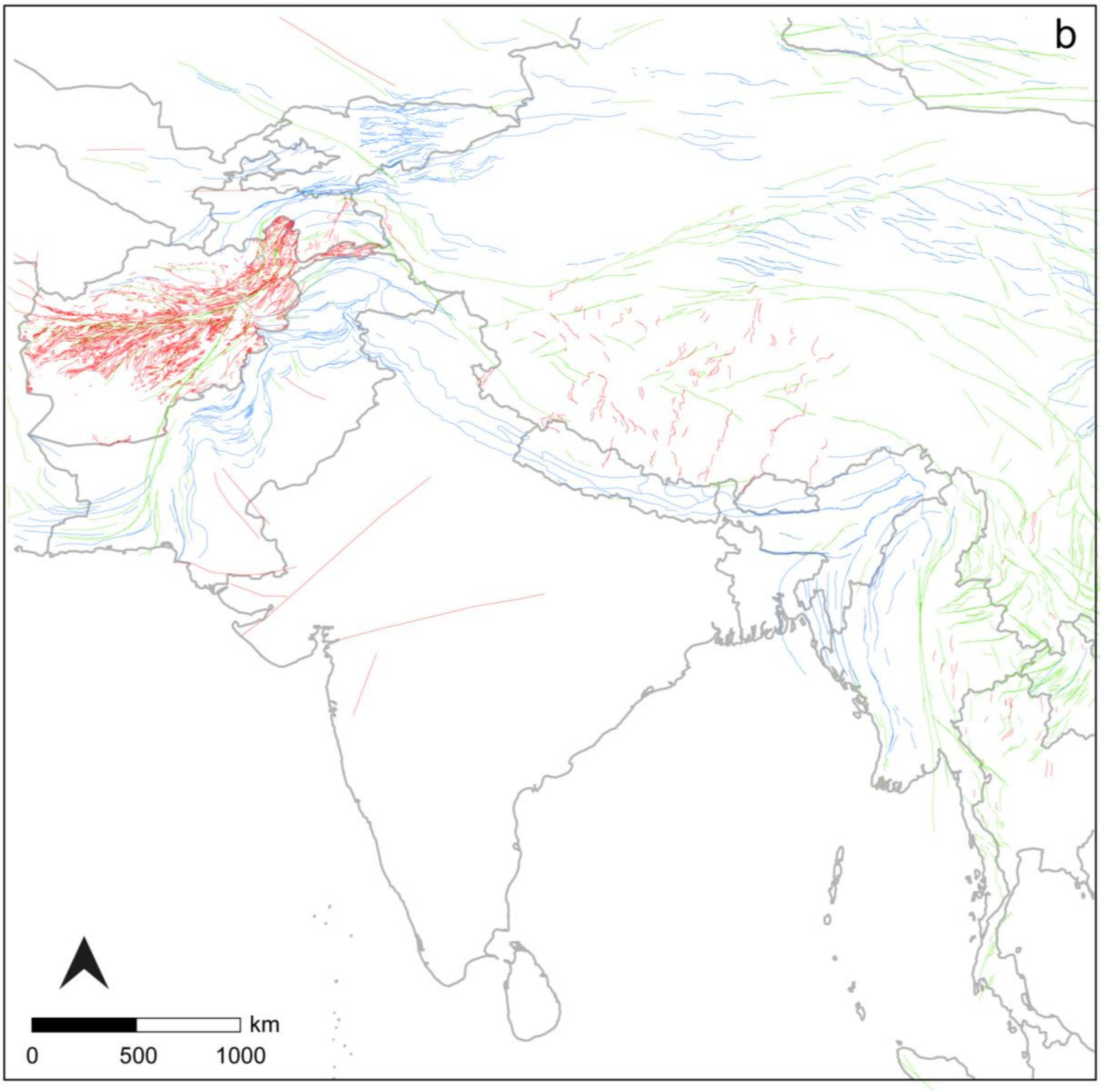
b
km
0
500
1000

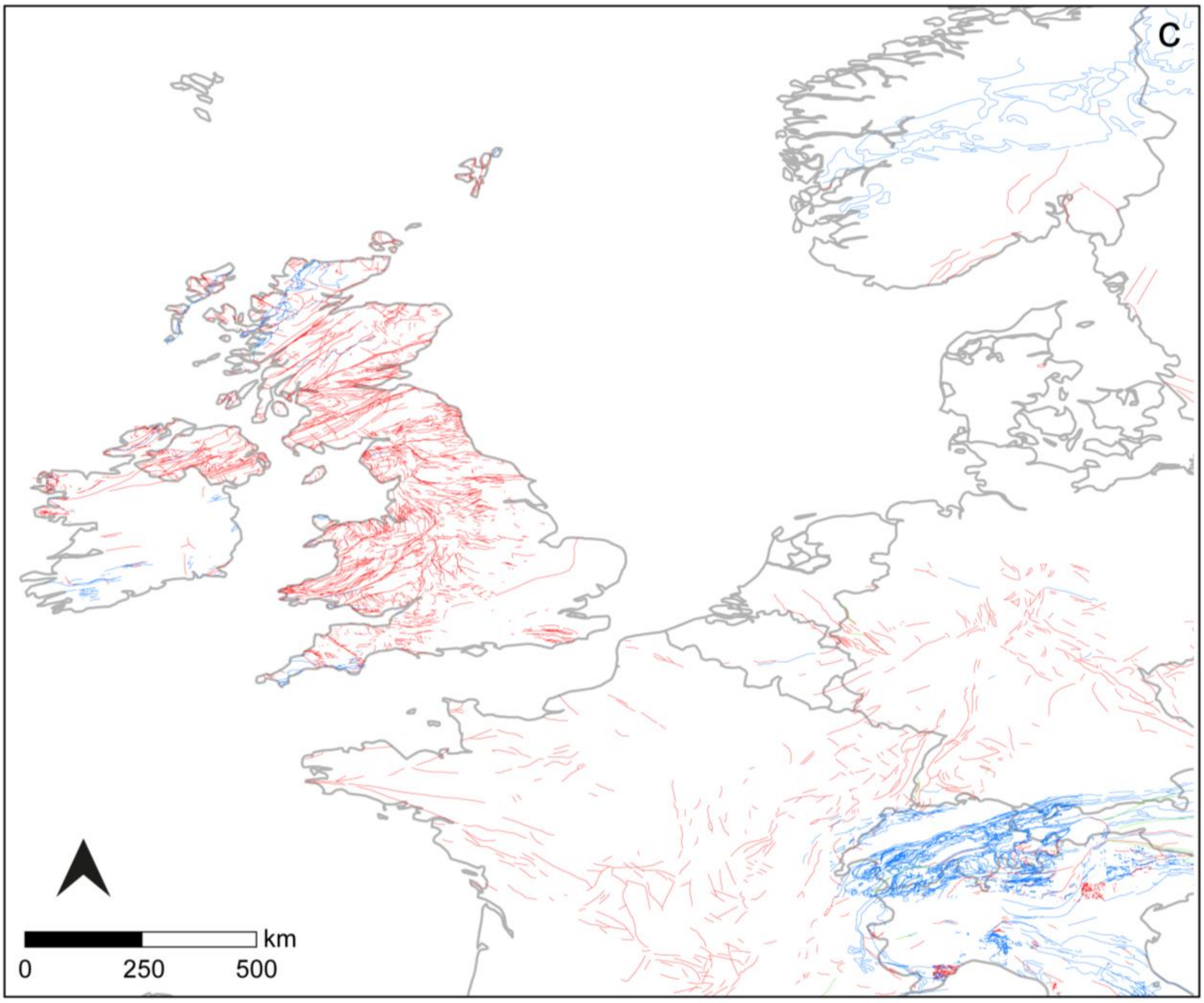
c
km
0
250
500

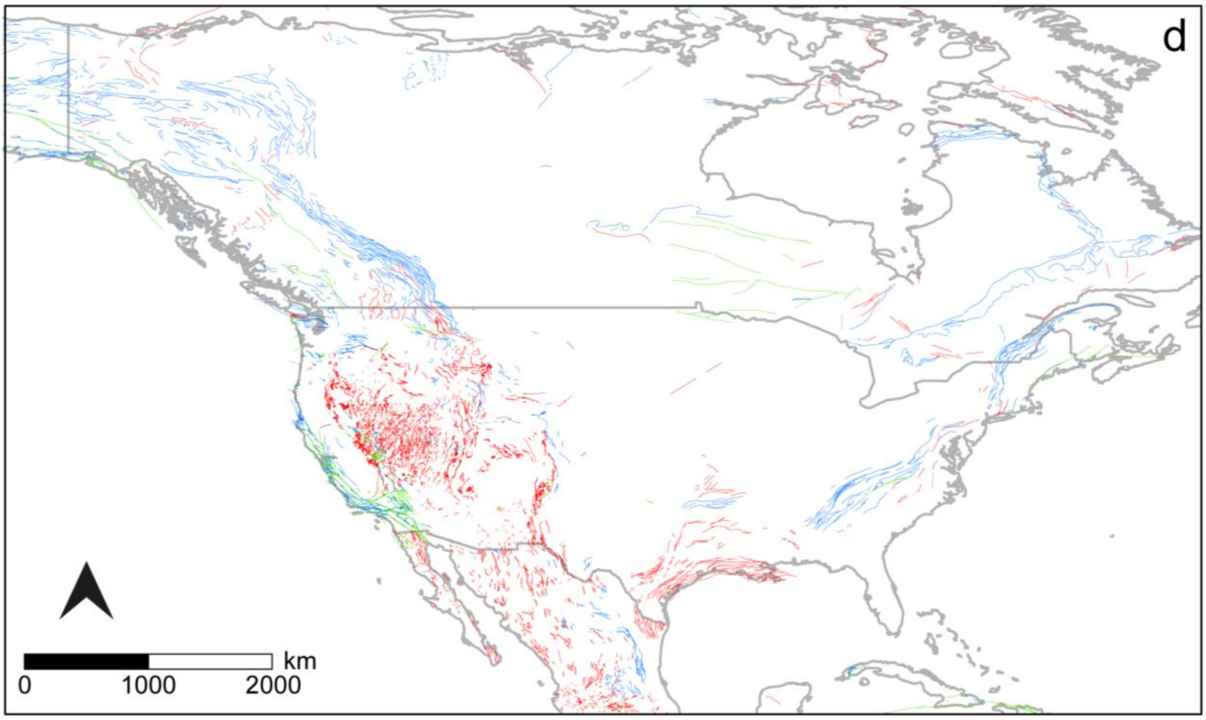
d
km
0
1000
2000

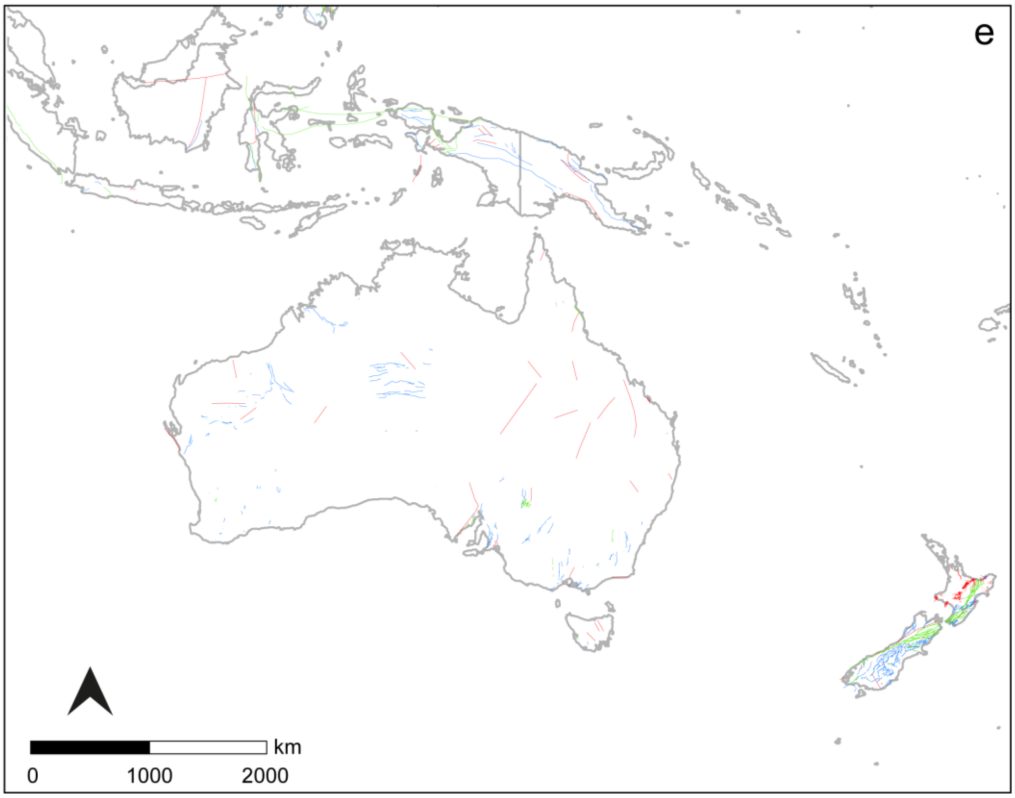

Supplementary Figure 7: Examples of regional maps extracted from the global fault dataset. (a) Southern Europe and Turkey; (b) Central Asia; c) North-Western Europe; d) Northern America; e) Australia. For the legend see Supplementary Figure 6.

The map of the global fault density was derived from the global fault dataset by using the kernel density algorithm[8] and represents the number of faults, weighted for their length (km) per km^2 (N_f km_f km^{-2}) (Supplementary Figure 8) (see Methods).

Fault density maps was also constructed for normal, strike slip and reverse style (Supplementary Figures 9a-b-c).

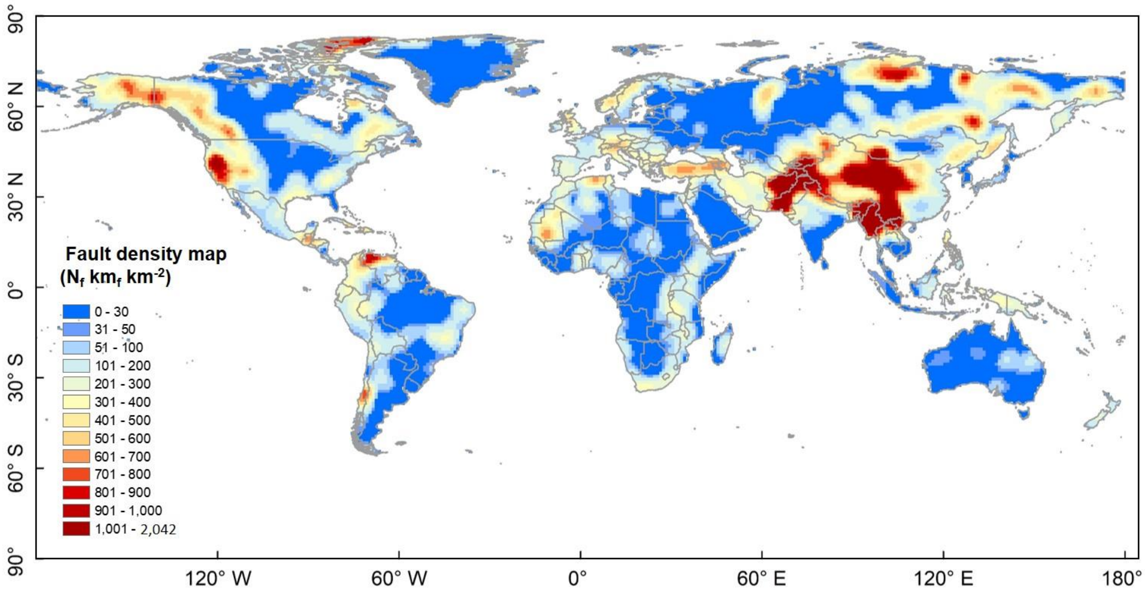

Supplementary Figure 8: Fault density map. The map was obtained by Kernel density algorithm (see Methods).

Relationship between seeps and fault style. We used the near distance tool of ArcGIS Pro to define the style (normal, strike slip, reverse) of the nearest fault to each seep. We stress that the nearest fault considered in this analysis serves solely as source of information on the faulting style associated with seepage, and does not necessarily represent the actual fault along which fluids migrated originating the seep.

Statistics of the distances between the seeps and the near fault is reported in Supplementary Table 2. Supplementary Table 2 shows that data are positive skewed. In this case, the arithmetic mean will fail to describe the "central tendency". Furthermore, the similarity between the median and the geometric mean suggests that the statistical distribution of the distances from the nearest fault follows a log-normal statistical distribution (i.e., the log-transformed data show a normal distribution). Accordingly, the geometric mean (GM) can be considered an unbiased estimator of the average value.

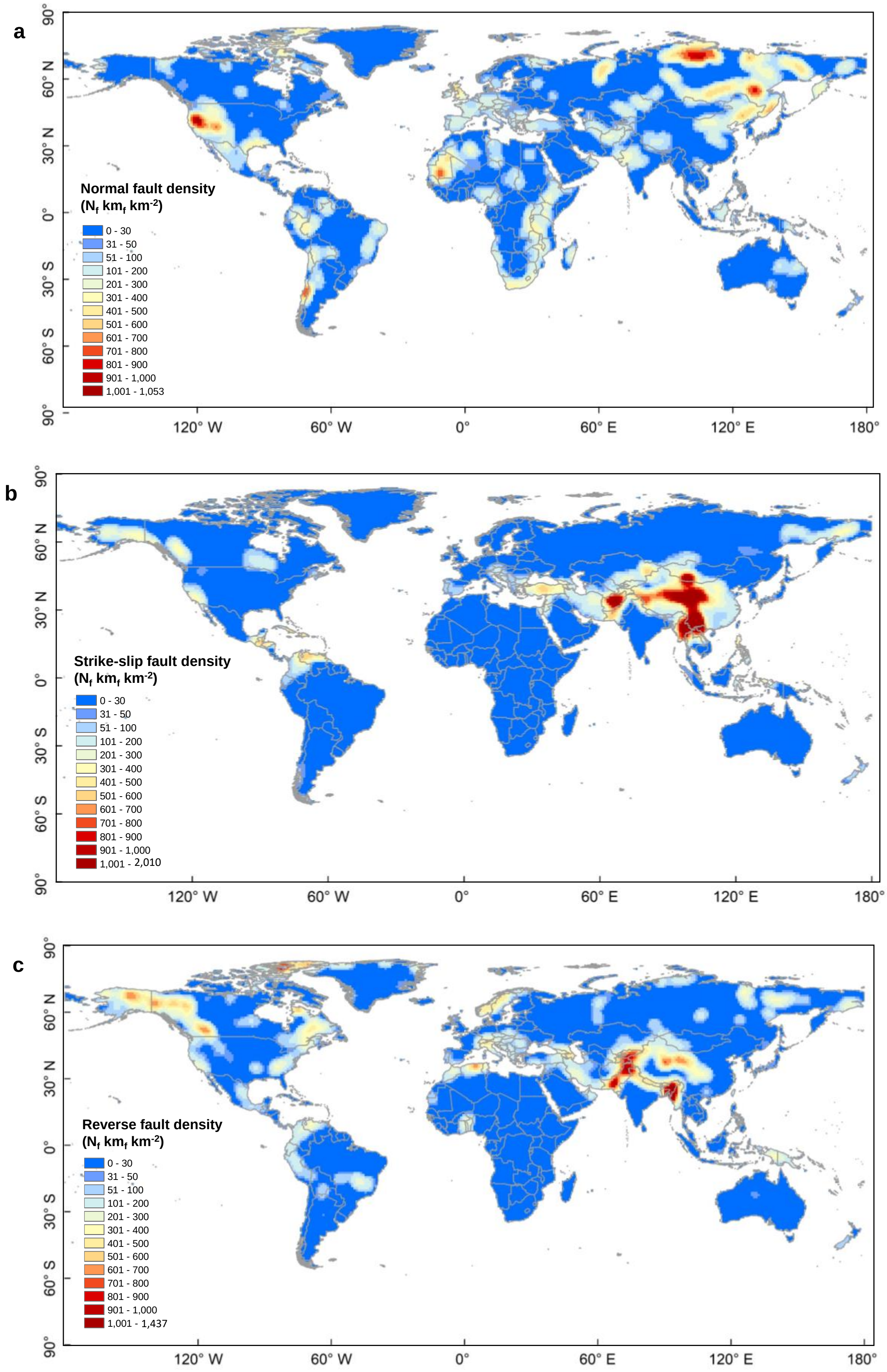

Supplementary Figure 9: Fault density map. (a) Normal, (b) strike slip and (c) reverse fault styles.

Supplementary Table 2: Statistics of the distances (km) between the seep types and the type of nearest fault system. GM: geometric mean; Min: minimum value; Max: maximum value; LQ: lower quartile; UQ: upper quartile; 5%: 5th percentile; 95%: 95th percentile; Std. Dev.: standard deviation.

Fault type	N	Mean	GM	Median	Min	Max	LQ	UQ	5%	95%	Std.Dev.
**Reverse**											
Oil seeps	691	26.2	3.6	3.6	0.0	582.7	1.0	17.9	0.1	107.7	75.9
Gas seeps	411	26.5	3.8	3.1	0.0	337.9	0.8	18.7	0.1	113.1	53.0
MV	514	66.4	17.0	13.6	0.0	411.4	6.3	56.5	0.6	364.0	111.9
**Strike Slip**											
Oil seeps	214	33.7	4.3	4.8	0.0	525.3	0.9	30.2	0.0	154.1	75.5
Gas seeps	67	72.6	11.4	9.1	0.1	523.3	2.1	72.7	0.3	451.3	130.2
MV	78	108.9	15.6	13.1	0.0	516.6	2.4	108.4	0.2	472.7	169.9
**Normal**											
Oil seeps	211	101.5	27.1	31.4	0.0	785.4	7.6	132.8	0.7	419.8	147.1
Gas seeps	360	76.8	37.7	75.9	0.0	528.7	31.6	89.1	0.7	256.8	75.2
MV	149	59.1	22.0	24.3	0.3	548.5	8.0	56.7	1.3	277.0	95.8

Histograms of Supplementary Figure 10 highlight that the majority of the seeps occur within the first 20 km from the nearest fault system and that seep number rapidly decreases beyond this distance.

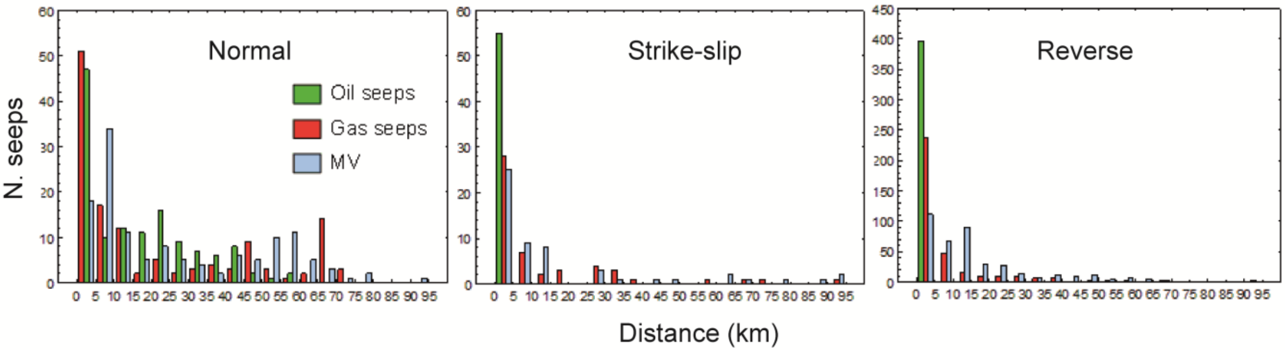

Supplementary Figure 10: Histograms of the seep distances from the nearest normal, strike slip and reverse faults.

Normal Probability Plots of Supplementary Figure 11 show that the majority of the seeps (about 60%) occur within 20 km from the nearest fault system bearing the information of the faulting style, which is within the uncertainty of geographic position of either seep and/or fault inventories.

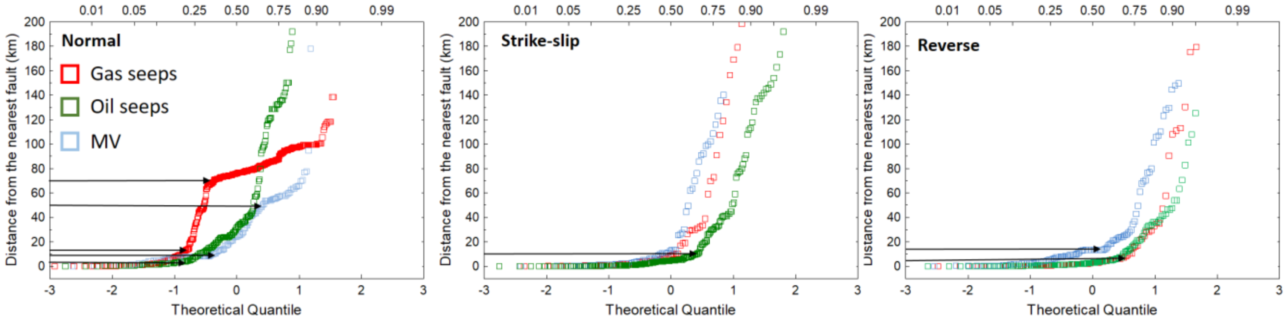

Supplementary Figure 11: Normal probability plots of seep distances from the nearest normal, strike slip and reverse fault. The plot highlights the presence of threshold distances at which statistical populations of seeps occur (black arrows).

Supplementary Table 3: Number of faults within petroleum field areas.

Fault type	N.
Normal	3155
Reverse	4585
Strike-slip	557

Supplementary Note 5 : Spatial modelling of geo-CH4 seepage favourability

The results of the geospatial analysis of the relationships between seeps and the four geological factors considered in this work (petroleum field area, type of sedimentary basins, heat flow, and faulting style) have been used to build a spatial model of seepage favourability. The model was developed using GIS-based MCDA (or Spatial Multi Criteria Decision Analysis, SMCDA) method[9].

The effective factors used to generate the geo-CH_4 seepage favourability map (1° x 1° grid) are expressed in the form of Boolean grid maps with Code 0/1 (areas excluded from consideration being coded with a 0 and those open for consideration being coded with a 1) or continuous grid values[9,10].

The ranges of suitability of seepage for each geological factor (called "constraints" in SMCDA)[9,11,12] resulting from the geospatial analysis are:

Petroleum field area (PFA): we found that the majority of the seeps (81%) occur in petroleum field areas and, therefore these areas constitute a typical habitat for seepage. Code 1 was then attributed to the existence of petroleum fields, and 0 to their absence.

Basins: Convergent basins (CB) host the majority of seeps (72%; Table 1) and thus they are a more favourable seepage habitat compared to divergent basins (28%). Code 1 was then attributed to convergent basins, and 0 to divergent basins (DB).

Heat flow (HF): the statistical analysis of heat flow values at seep locations highlights that 95% of the seeps occur in areas with heat flow < 98 mW m^{-2} (Supplementary Table 1). Code 1 was then attributed to areas with heat flow below 98 mW m^{-2} and code 0 to areas with above 98 mW m^{-2}.

Faults: Fault density grid maps for the three different fault types (continuous values) were used to define constraints for seepage. The maps are reclassified by using code 1 for the fault density values in which most of the seeps occur and 0 for the other values according to the results shown by the histogram in Figure 2b. In particular, for reverse faults (RF) code 1 is attributed to density values in the range 100-600 N_f km_f km^{-2}, for strike slip faults (SSF) code 1 is attributed to density values in the range 100-800 N_f km_f km^{-2} and for normal faults (NF) code 1 is attributed to density values in the range 0-400 N_f km_f km^{-2}.

Using the Analytical Hierarchy Process (AHP; the first step of SMDCA)[11,13] the four geological factors have been compared to evaluate their relative importance (Supplementary Table 4) in order to construct a 7 x 7 pairwise comparison matrix as reported in Supplementary Table 5. The relevance attributed to each factor was ranked on a ratio scale based on expert judgment (a typical approach of AHP).

Supplementary Table 4: Pairwise comparison of the factors. The selected values are in bold.

Factors	Extreme favours	Very strong favours	Strongly	Sligthly	Equal	Sligthly	Strongly	Very strong favours	Extreme favours	Factors
HF	9	7	5	**3**	1	3	5	7	9	CB
HF	9	7	**5**	3	1	3	5	7	9	DB
HF	9	7	5	3	**1**	3	5	7	9	PFA
HF	9	7	5	**3**	1	3	5	7	9	RF
HF	9	**7**	5	3	1	3	5	7	9	SSF
HF	9	**7**	5	3	1	3	5	7	9	NF
CB	9	7	**5**	3	1	3	5	7	9	DB
CB	9	**7**	5	3	1	3	5	7	9	PFA
CB	9	7	5	3	**1**	3	5	7	9	RF
CB	9	7	5	**3**	1	3	5	7	9	SSF
CB	9	7	5	**3**	1	3	5	7	9	NF
DB	9	7	5	3	1	3	5	7	**9**	PFA
DB	9	7	5	3	1	3	5	**7**	9	RF
DB	9	7	5	**3**	1	3	5	7	9	SSF
DB	9	7	5	**3**	1	3	5	7	9	NF
RF	9	7	**5**	3	1	3	5	7	9	SSF
RF	9	7	5	3	1	3	**5**	7	9	PFA
RF	9	7	**5**	3	1	3	5	7	9	NF
SSF	9	7	5	3	1	**3**	5	7	9	NF
SSF	9	7	5	3	1	3	5	7	**9**	PFA
NF	9	7	5	3	1	3	5	**7**	9	PFA

The relative importance of the geological factors reported in Supplementary Table 4 was then transformed into a ratio/number matrix (Supplementary Table 5a, b). The AHP assigned factor weights by summing the values in each column of the ratio/number matrix[11,13]. The matrix is then normalized by dividing each element by its column total, and finally averaging the elements in each row of the normalized matrix (Supplementary Table 6). This procedure is the best way to minimise the impact of inconsistencies in the ratios[11]. The calculated weights of the factors are shown in the last column of Supplementary Table 6.

Supplementary Table 5a: Pairwise comparison matrix as ratio.

	HF	CB	DB	PFA	RF	SSF	NF
**HF**	**1**	3	5	1	3	7	7
**CB**	1/3	**1**	5	1/7	1	3	3
**DB**	1/7	1/7	**1**	1/9	1/7	1/3	1/3
**PFA**	1/3	5	9	**1**	3	9	7
**RF**	1/5	1/5	5	1/5	**1**	5	5
**SSF**	1/9	1/5	3	1/9	1/7	**1**	1/3
**NF**	1/9	1/5	3	1/7	1/5	3	**1**
**Column total**	**2.098**	**9.876**	**31.000**	**2.708**	**8.486**	**28.333**	**23.667**

Supplementary Table 5b: Pairwise comparison matrix as number.

	HF	CB	DB	PFA	RF	SSF	NF
**HF**	1.000	3.000	5.000	1.000	3.000	7.000	7.000
**CB**	0.333	1.000	5.000	0.143	1.000	3.000	3.000
**DB**	0.143	0.143	1.000	0.111	0.143	0.333	0.333
**PFA**	0.333	5.000	9.000	1.000	3.000	9.000	7.000
**RF**	0.200	0.333	5.000	0.200	1.000	5.000	5.000
**SSF**	0.111	0.200	3.000	0.111	0.143	1.000	0.333
**NF**	0.111	0.200	3.000	0.143	0.200	3.000	1.000
**Column total**	**2.098**	**9.876**	**31.000**	**2.708**	**8.486**	**28.333**	**23.667**

Supplementary Table 6: Normalised matrix and final weights.

	HF	CB	DB	PFA	RF	SSF	NF	Weight
**HF**	0.477	0.304	0.161	0.369	0.354	0.247	0.296	**0.315**
**CB**	0.095	0.101	0.161	0.053	0.118	0.106	0.127	**0.109**
**DB**	0.068	0.014	0.032	0.041	0.017	0.012	0.014	**0.028**
**PFA**	0.159	0.506	0.290	0.369	0.354	0.318	0.296	**0.327**
**RF**	0.095	0.034	0.161	0.074	0.118	0.176	0.211	**0.124**
**SSF**	0.053	0.020	0.097	0.041	0.017	0.035	0.014	**0.040**
**NF**	0.053	0.020	0.097	0.053	0.024	0.106	0.042	**0.056**

The model highlights the high importance of the petroleum field areas and of the heat flow (weights 32.7% and 31.5% respectively), and subordinately of reverse faults and convergent basins (weights 12.4% and 10.9% respectively). Because the comparisons were carried out through subjective judgement, some degree of inconsistence and bias may occur. To guarantee a confidence level of consistency throughout, the Consistency Index (CI) and the Consistency Ratio (CR) are used to perform a consistency verification of the pairwise matrix[13,14]. CI and CR are 0.0019 and 0.0014, respectively; at CR < 0.1 the pairwise comparison matrix is considered consistent[14]. The weighted linear combination (WLC) of the raster maps of the considered geological factors have been obtained multiplying the maps by their respective calculated weights and then summing the products to obtain the final map of the percentage of favourability (see main text Fig. 4a) (Supplementary Figure 12).

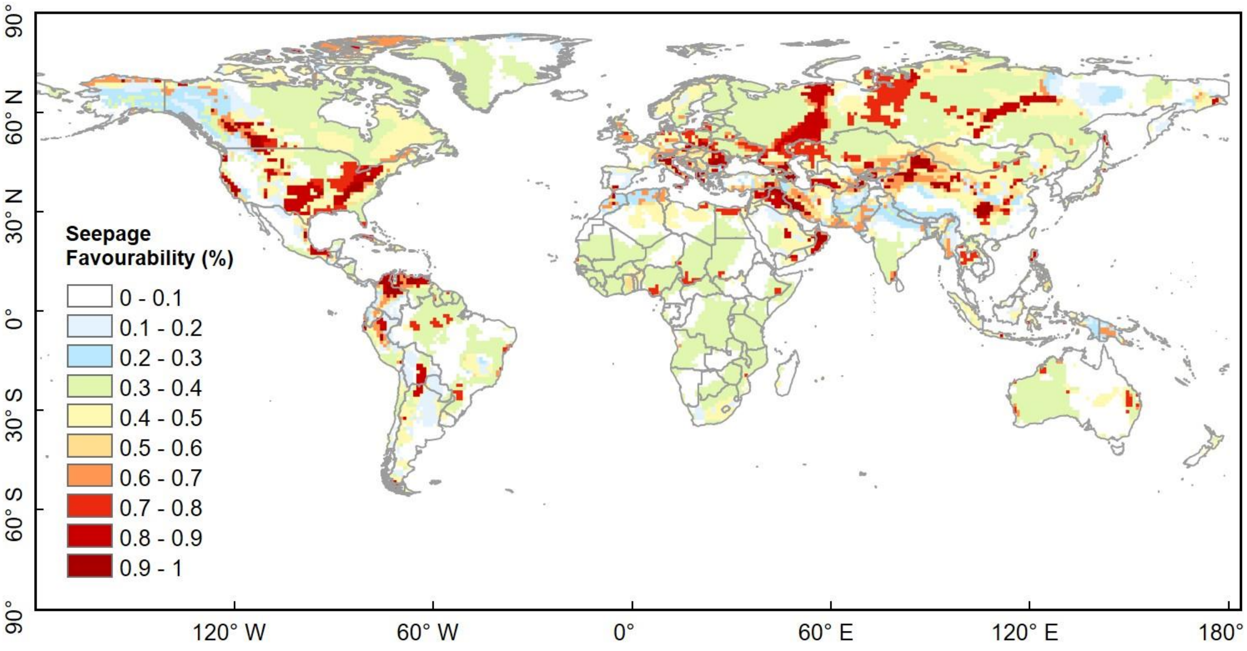

Supplementary Figure 12: Map of seepage favourability.

The number of known (inventoried) seeps within each decile class was calculated to validate the seepage favourability. A rapid increase of the number of seeps occur in cells with favourability higher than 50% (about 87% of the seeps) (see main text Fig. 4b).

The favourable areas consist of 2516 1x1° grid cells, 1558 (62%) occur in convergent basins and 881 (32.5%) in divergent basins, respectively. The remaining 5.5% (138 seeps) occur in areas not covered by basins. In particular, the largest favourable areas occur in the following areas:

- North America (Interior Platform, Intracratonic Sag, Foreland, Appalachian and North America Rockies basins, Convergent Basins),
- Western Russia Peripheral Foreland basins and Caucasus Retroarc (Convergent Basins) and Eastern European Rift Intracratonic Sag (Divergent Basins)
- Western Siberia and European Arctic post-rift sag basins (Divergent basins)
- East Siberia Vilyuy Rift (Peripheral Foreland, Convergent Basins) and Patom Baykit (Intracratonic Sag, Divergent Basins)
- Eastern Paratethys (Qilian Shan Foreland, Tien Shan and Mid China Peripheral Foreland, and Pacific Margin Rift, Convergent Basins)
- Central Paratethys, Turkmenistan, Kyrgyzstan (Peripheral Foreland, Convergent Basins)
- Himalayan Peripheral Foreland (Convergent Basins)
- Central Myanmar Retroarc Foreland and Himalayan Peripheral Foreland (Convergent Basins) and Indo-Burma Forearc (Divergent Basins)
- Arabian Peripheral Foreland (Convergent Basins)
- Western Paratethys, Northern Carpatians (Retroarc Foreland, Convergent Basins) and North-Eastern Europe (Post-rift Sag, Divergent Basins)
- Western Paratethys – Western Europe (Circum-Adriatic Foreland, Peripheral Foreland Convergent Basins)

Supplementary References

1. Macgregor, D.S. Relationships between seepage, tectonics and subsurface petroleum reserves. *Mar. Pet. Geol.*, **10**, 606-619 (1993).
2. Etiope, G. *Natural Gas Seepage, The Earth's Hydrocarbon Degassing*, pp. 199 (Springer, Switzerland, 2015).
3. Abrams, M.A. Significance of hydrocarbon seepage relative to petroleum generation and entrapment. *Mar. Pet. Geol.*, **22**, 457-477 (2005).
4. Mazzini, A. & Etiope, G. Mud volcanism: an updated review, *Earth Sci. Rev.*, **168**, 81-112 (2017).
5. Link W.K. Significance of Oil and Gas. Seeps in World Oil Exploration. *AAPG Bulletin*, **36**, 1505-1541 (1952).
6. Etiope, G., Ciotoli, G., Schwietzke, S. & Schoell, M. Gridded maps of geological methane emissions and their isotopic signature. *Earth Syst. Sci. Data*, **11**, 1-22 (2019).
7. IHFC. The Global Heat Flow Database of the International Heat Flow Commission. Available at: [http://www.datapages.com/gis-map-publishing-program/gis-open-files/global-framework/global-heat-flow-database] (2016).
8. Silverman, B. W. *Density Estimation for Statistics and Data Analysis*. (New York: Chapman and Hall, 1986).
9. Malczewski, J. *GIS and Multicriteria Decision Analysis*, pp. 408 (John Wiley and Sons, New York, 1999).
10. Chen, K, Blong R. & Jacobson, C. MCE-RISK: Integrating multicriteria evaluation and GIS for risk decision-making in natural hazards. *Environ. Modell. Softw.*, **16**, 387-397 (2001).
11. Saaty, R. W. The Analytic Hierarchy Process - What It Is and How It Is Used. *Math. Model.*, **9**, 161-176 (1987).
12. Malczewski, J. GIS-based multicriteria decision analysis: a survey of the literature. *Int. J. Geogr. Inf. Sci.*, **20**, 703-726 (2006).
13. Saaty, T. Decision Making with the Analytic Hierarchy Process. *Int. J. Serv. Sci.*, **1**, 83-98 (2008).
14. Saaty, T.L. *Fundamental of Decision Making and Priority Theory with AHP*. RWS Publications, Pittsburgh, PA, USA (1994).
